# The Role of the Social Environment in Pacing and Sports Performance: A Narrative Review from a Self-Regulatory Perspective

**DOI:** 10.3390/ijerph192316131

**Published:** 2022-12-02

**Authors:** Kandianos Emmanouil Sakalidis, Stein Gerrit Paul Menting, Marije Titia Elferink-Gemser, Florentina Johanna Hettinga

**Affiliations:** 1 Department of Sport Exercise and Rehabilitation, Faculty of Health and Life Sciences, Northumberland Building, Northumbria University, Newcastle upon Tyne NE1 8ST, UK; 2 Department of Human Movement Sciences, University Medical Center Groningen, University of Groningen, P.O. Box 196, 9700 AD Groningen, The Netherlands

**Keywords:** metacognition, self-control, motivation, coaches, opponents, athletes

## Abstract

As proposed by Triplett in 1898 and evidenced by a recent series of lab and field studies, racing against other competitors consistently results in increased performance compared to when racing alone. To explain this phenomenon, we will explore the process of self-regulation, a process relevant to pacing, which is linked to athletes’ emotions and facilitates their sports performance optimization. We will apply the cyclical model of Self-regulation of Learning to pacing and sports performance settings and explore the role of the social environment (in particular, opponents but also coaches) in each phase of the self-regulatory model. It seems that the social environment could be considered as a significant self-regulatory and sports performance facilitator. More specifically, athletes can focus on their social environment (opponents) when they have to set goals and select appropriate strategies to achieve them (forethought phase), monitor and manage their actions and their emotions (performance phase), and make self-judgements and choose self-reactions (self-reflection). Moreover, the social environment (coaches) can observe, step in, and facilitate these intricate processes. These findings could guide athletes and their coaches towards more effective pacing acquisition and development, and better sports performance, which could be of particular relevance for youth athletes or athletes with disabilities impacting on their self-regulatory skills.

## 1. Introduction

Pacing has been described as a goal-directed process of decision making in which athletes decide when and how to distribute their limited energy resources throughout an exercise task [1,2]. Adequate pacing behaviour is critical in sports as it facilitates optimal performance [3,4]. In sports settings, numerous lab and field studies consistently revealed that the social environment is an influential factor in athletes’ pacing and performance [5,6,7,8,9,10,11], as first observed by Triplett [12]. In head-to-head sports, such as cycling, running, and speed skating, opponents can act as social placebos, inviting changes in athletes’ pacing behaviour [6,7,10] impacting positively on performance [8,13,14], while their rate of perceived exertion remains the same [15]. According to the ecological perspective, this occurs because opponents act as social affordances (invitations for actions), providing the athlete with additional performance feedback and therefore influencing their decision-making [2,6,15]. This suggests that when coaches and athletes use the environment and its opportunities to provide performance feedback smartly, this could enhance training as well as competitive performance. To understand how this can be conducted, we will explore pacing and racing against opponents from a self-regulatory perspective, in which learning from previous feedback is a central component. This approach will also allow us to incorporate the role of emotions in competition.

The pacing behaviour of athletes can be characterised as a self-regulatory process of energy regulation [16]. To be more specific, self-regulation commonly refers to cognitive and behavioural processes that facilitate behaviour adaptation. It is essential to a person’s functioning and an early marker of life success [17]. Central to self-regulation is the learning process, as proposed by the framework of Self-Regulation of Learning (SRL), whereby individuals are responsible for their own learning. In this learning process, individuals have to acquire and adapt self-regulatory skills to overcome challenges and attain behaviour goals [18]. Thus, the concept of SRL is relevant to pacing because skills such as planning, monitoring, and evaluating are thought to underpin the cyclical process of refining the performance template underpinning pacing behaviour [16]. By cycling through this process, the athletes’ pacing behaviour is adapted to the task demands (e.g., task duration, biomechanics, and environmental factors) resulting in improved exercise task performance [16,19]. The self-regulatory processes of pacing could be influenced by opponents as previous studies suggest [8,13,14], but this has not been explored in depth.

Indeed, the self-regulatory perspective seems to provide a promising framework for studying both the development and optimalization of pacing behaviour and performance [16]. However, the few studies that have tried to integrate the SRL framework within the pacing and sports performance settings emphasise the role of individuals’ efforts in their own self-regulatory process [16,20,21]. It is not well documented how athletes can use their opponents as an influential factor in enhancing self-regulatory skills, which are important in pacing. This is remarkable considering the influence of the social environment in developing and optimizing athletes’ pacing behaviour [6,19]. The further investigation of the relationship between self-regulatory skills, pacing, and the opponents could inform coaches about the appropriate strategies that they should follow to enhance athletes’ pacing optimisation in training and competition and guide the development of athletes [16]. It could also advise athletes how to appropriately react to opponents’ actions, regulate their emotions, and pace themselves. In view of the above, the purpose of this narrative review is to explore why and how we should consider the social environment in SRL in the context of pacing behaviour and sports performance.

Based on all the above, the aims of this review are: (1) to apply the cyclical model of Self-regulation of Learning to pacing and sports performance settings and (2) to explore the role of the opponents and coaches in each phase of the self-regulatory model (forethought, performance, and self-reflection phase). This approach could provide in-depth information and novel insights with impact on the further development of interventions, coaching and guidance towards pacing acquisition and development, and optimal performance. It could also inform athletes how to use their social environment to regulate their actions, emotions, and pace and perform better.

## 2. The Cyclical Model of SRL

The Cyclical Model of SRL describes the interrelation between the cognitive, metacognitive, and motivational mechanisms in three self-regulatory phases: forethought, performance, and self-reflection [18]. The forethought phase consists of task analysis and self-motivation processes [18]. Task analysis involves two parts: setting goals (the outcomes that an individual wants to attain) and strategic planning (choosing learning methods that are appropriate for the task). The forethought phase is influenced by different sources of self-motivation, such as outcome expectations (believed consequences of an individual’s behaviour), goal orientation (reasons for engaging in specific behaviours), intrinsic interest/intrinsic motivation (the individual’s liking or disliking of an activity due to intrinsic motives) and self-efficacy (individual’s beliefs in their abilities to think and act towards their learning goals) [22,23].

The performance phase follows the forethought phase and is composed of self-control and self-observation methods [18]. Individuals use a variety of strategies to attain self-control such as imagery (forming mental pictures to facilitate the learning process), help-seeking (providing assistance when an individual performs an activity) and self-consequences (setting rewards or punishments). However, these strategies need to be adapted based on the individual’s learning goals. Thus, self-observation is considered a critical process that guides individual’s self-control to perform an activity. Self-observation consists of two processes: self-monitoring (mental tracking of the individual’s performance) and self-recording (creating records of outcomes and learning processes).

The self-reflection phase is the last of the three phases and consists of two processes: self-judgements and self-reactions [18]. Self-judgements involve comparisons of an individual’s performance with a standard such as prior performance, social comparison with others, or expert mastery. Self-reactions are composed of self-satisfaction (affective and cognitive reactions to the individual’s self-judgements) and adaptive/defensive decisions (individual’s willingness to further engage in the learning process or not). People who decide to further engage in the learning process, plan, monitor, and evaluate their actions based on the previously gathered experiences. It is worth mentioning that the Cyclical Model of SRL pays special attention to the reciprocal and dynamic interaction of the individual’s behaviour and the environment (reciprocal determinism) [24]. In other words, individuals learn to self-regulate through social means such as social support, feedback, and modelling (observational learning) [18,24]. 

## 3. The Integration of Pacing and Sports Performance within the Cyclical Model of SRL

The cyclical model of SRL [18] seems to be applicable in elite performance sports. Successful athletes have to take responsibility of their own training and learning procedures over a period of multiple years and take appropriate actions to improve their pacing behaviour and stay motivated throughout the process [16]. To gain a better understanding of the self-regulatory aspects of pacing, this study will try to integrate different performance periods within the three phases of the cyclical model of SRL, similarly to Elferink-Gemser and Hettinga [16]. More specifically, we will first examine the forethought phase of the cyclical model of SRL, which is the period where individuals plan their appropriate pacing strategy in training (before competition; training period). During this phase, athletes should be able to set goals (e.g., keep a set pace for the first half of a 10 km race; pacing strategy) and plan how they will attain them (e.g., additional training sessions, twice a week) [16,18]. Subsequently, during the performance phase, athletes compete in an event (during competition), and they try to attain self-control through imagery (e.g., runners imaging themselves to appropriately distribute their energy during the race by taking into consideration the competitors’ actions), help-seeking (e.g., basketball players asking instructions from a coach to improve their ball possessions’ frequency during a game), and self-consequences (e.g., setting a reward for a pacing accomplishment). More importantly, athletes monitor and self-record their effort exertion, use numerous strategies to keep themselves motivated and adapt their effort exertion to optimally reach the exercise task goal [16,18]. Lastly, the self-reflection phase could be considered as the period after the participation of athletes in competition. After a competition, athletes judge their pacing behaviour based on specific standards (e.g., outcome of the competition, comparison with opponents or other athletes) and react accordingly [16,18]. On the basis of their self-judgements, athletes decide if they will continue to engage, adapt, and improve their pacing (adaptive decisions) or if they will avoid further efforts to learn and improve (defensive decisions) [25]. When the athlete decides to continue engaging, the goals are set based on the experience gathered during and after competition, restarting the cycle with the forethought phase. Thus, it is not surprising that high scores on self-reflection are associated with a higher level of performance, as elite athletes are more aware of their strengths and weaknesses in performance settings [26,27]. On the basis of all the above, and with a purpose to provide pacing-related guidelines to the coaches, this study will try to explore the impact of opponents within these three phases.

## 4. Social Environment, Pacing, and Sports Performance within the Cyclical Model of SRL

### 4.1. Forethought Phase

The social environment could be a noteworthy self-regulatory, pacing, and sports performance facilitator during this phase. It offers multiple opportunities in order to guide and assist athletes to set realistic performance goals before a competition. For instance, athletes can use opponents as goal setters, as their presence and/or their pacing behaviour could influence their goal setting and motivate them to engage in a specific pacing behaviour (e.g., athletes are planning how to optimise their pacing behaviour to perform better than their opponents) [28]. Athletes who are more oriented towards wins and losses (e.g., beating an opponent) plan their pacing behaviour and performance less appropriately compared to athletes who are more focused on their personal goals [29]. For example, better competitors could lead them to adopt unrealistic and/or ineffective pacing and performance goals [28,30], a finding that athletes and their coaches should take into consideration during the training period. 

During the forethought phase, coaches have an important role in the facilitation of athletes’ goal setting and strategic planning, two self-regulatory skills which are relevant to pacing. For instance, coaches’ goals can influence the goals that athletes set for themselves [31]. Moreover, coaches who set more challenging goals (e.g., beat an elite athlete) and have higher expectations from their athletes have a positive impact on their performance [32]. It seems though, that setting of training and competition goals (short- and long-term goals) along with the coach (co-orientation) also plays an important role in improving athletes’ skills, and potentially pacing, as it keeps them motivated and focused (e.g., their common goal is for the athlete to respond better to external factors such as the opponent during competition) [33,34]. When coaches and athletes are setting goals (e.g., based on the pacing behaviour of their opponents), special attention should be paid to athletes’ personality. Athletes with a more internal locus of control (control over the events that influence peoples’ lives) spend more time on-task when they set their own goals (self-set goals). Athletes with a more external locus of control (events that happen in peoples’ lives are out of their control) spend more time on-task when coaches set their goals, and these goals could be dictated by their opponents’ behaviour and/or abilities [35]. Thus, it might be more beneficial for coaches who coach athletes with external locus of control, to set their athletes’ goals based on their opponents’ behaviour/abilities. This mental visualisation of the goal could act as a motivational factor during the forethought (and later at the performance) phase [35].

To optimally develop and guide athletes’ pacing, coaches can play a major role and help athletes to strategically plan their activities and empower them in making the right decisions at the right time, particularly in relation to energy distribution, training load distribution across a training period, and general fatigue and recovery management [36,37,38]. Coaches who want to facilitate the strategic planning process should give a lot of emphasis on how they can assist athletes to anticipate potential obstacles in competition (e.g., anxiety) and empower them to be prepared for future barriers (e.g., athlete has to compete against faster paced opponents) [37]. In order to engage athletes in the strategic planning process, coaches devote a lot of time to support athletes’ motivational beliefs (motivation and self-efficacy, e.g., opponents can be used as external motivators), as this type of focus would lead athletes to their desired outcome [37]. These behaviours are in line with the findings of Goffena and Horn [38], where there is a relationship between autonomy-supportive coaching and athletes’ strategic planning.

### 4.2. Performance Phase

During sports competition, athletes are constantly looking for information in order to monitor their pacing behaviour and achieve the specific performance goals that were established at the forethought phase. Their interaction with the opponents could facilitate these processes. Opponents could be characterised as the visual representation of a goal throughout the race, hereby providing direct feedback to the athletes about the progress of the competition and their own pacing behaviour and performance [9,13,15,39,40]. Thus, it is not surprising that the attention of athletes (with a purpose to monitor their actions) mainly focuses on the opponent, especially where there is a high interdependency between them [13,39,41,42,43]. For example, when athletes are experiencing a positive momentum in relation to their opponents, they are able to regulate more efficient their exercise intensity within the race compared to a negative momentum [44]. Another interesting finding is that, even if athletes performed significantly faster in head-to-head (against an opponent) compared to time trial (no opponents) cycling and running trials, athletes rated their perceived effort similarly in both conditions [9,39]. This finding could be explained by the notion that the opponents function as an external distraction that decreases the athletes’ perceived exertion. During this phase, the opponents are the visual representation of a goal throughout the race and act as a motivational factor, shift athletes’ attentional strategies, and influence their decision-making [45]. Thus, when athletes compete against others, they can better regulate their afferent feedback, their pain, and fatigue, information that seems critical in pushing the limits of athletes’ sports performance [9,39,45].

In addition to all the above, the presence and the perception of the opponent during competition could play a role in athletes’ decision-making process and self-control ability, as the behaviour of an opponent affects the actions of an athlete and acts as a help-seeking factor [8,40,46,47,48,49]. For instance, a faster starting opponent evoked a faster start compared to a slower starting competitor in 4 km cycling time trials [8]. Moreover, Zouhal et al. [47] observed that drafting behind a runner improved the athletes’ performance in a 3 km trial, even in the absence of any physiological benefits. This finding indicates that following another runner may lead athletes to engage less in the decision-making process, a situation that decreases their cognitive loading (less depleting) and facilitates a more efficient self-control of their pacing during the competitive trial.

Coaches should take into consideration the important role of opponents on pacing and performance and prepare their athletes by engaging them in various competitive activities (against an opponent/s) that provide similar constraints in decision-making [8,14,47]. Due to the nature of sports competition, in this phase it may be more challenging for coaches to influence athletes’ behaviour and their ability to monitor their actions [5,50,51,52,53,54,55]. However, the provision of motivational feedback and encouragement by the coach could alter athletes’ activity monitoring, improve their attention, increase their objective performance, and may lead to a more appropriate pacing behaviour and improved sports performance outcome [5]. For example, external encouragement in both endurance and sprint trials resulted in improvements in athletes’ performance (faster pace), but they also reported significantly higher perceived exertion levels compared to the control trials (no external encouragement) [50]. Additionally, as opponents could alter athletes’ attention and improve their tolerance of fatigue [9,39], coaches’ feedback could focus more on the opponents’ actions and behaviour so they improve athletes’ motivation, pacing behaviour, and performance. 

There is also some evidence about the role of the coaches in athletes’ self-control [54,55,56]. For instance, Englert et al. [55] demonstrated that athletes who engaged in autonomously motivated self-control performed better under pressure compared to athletes who engaged in extrinsically motivated self-control. This finding indicates that autonomously motivated self-control is less depleting and gives an advantage to athletes under high pressure situations [55]. Except for autonomously exerted self-control, imagery instructions provided by the coaches (method of self-control) could guide athletes towards an external focus of attention (e.g., opponents-related instructions) [56], a finding that could have potential application in pacing. Coaches who focus on external cues (e.g., opponents) could alter athletes’ decision-making, facilitate their self-control abilities, and influence athletes’ drafting, positioning, and packing behaviour during a head-to-head trial [8,47,56].

### 4.3. Self-Reflection Phase

In sports settings, the social environment plays a significant role in the self-judgements’ and self-reactions’ process of athletes through their comparison with opponents and their affective reactions [57,58,59,60]. At a situational level, especially in a competitive environment where direct comparison can become salient, it is not surprising that the athlete may be susceptible to judging competence, evaluating success and failure, and continue engaging in the learning process (restarting the cycle with the forethought phase) based on social comparisons (e.g., outperforming an opponent due to a more efficient pacing behaviour at the beginning of the race) [57,58]. These social comparisons could also influence athletes’ affective reactions and cause athletes to experience positive or negative emotions towards their next performance [61]. The consequences of social comparison are interesting for pacing, as affective feelings are positively and/or negatively influenced by the opponents during a head-to-head trial and could alter athletes’ performance [62,63]. For instance, racing against an opponent could cause early fatigue (due to high intensity) and negatively influence athletes’ affective feelings [62,63]. Additionally, athletes experience more tension/pressure when they compete in groups compared to individual or one vs. one race [28]. The opponents’ pacing abilities could also play a role in athletes’ affective responses [63]. When athletes compete against a slower opponent, their affective feelings are increasing in the final stages of a 10 km cycling trial, which may be linked to increased certainty with regards to goal achievement [63]. Thus, through social comparisons opponents could play a significant role in the self-reflection phase and influence the further engagement of athletes in the pacing skills acquisition process [62,63].

Coaches could affect athletes’ self-reactions and promote their self-satisfaction (willingness to further engage in the pacing acquisition) through the facilitation of their affective reactions [64,65]. With a purpose to regulate their affective responses, coaches could focus on their athletes’ superiority over the opponent when athletes faced an inferior or equal opponent (lateral or downward comparison). In case of a superior opponent (upward comparison) though, coaches could focus on realistic expectations and pay more attention to athletes’ personal improvement [66]. Furthermore, there is an emotional connection between coaches and athletes. Coaches’ expressions of happiness predict athletes’ positive emotions (experience happiness) and could be associated with more appropriate pacing and better performance [67]. These positive affective reactions could increase the athletes’ willingness to develop their pacing based on their experiences gathered before, during, and after competition and facilitate their maintenance in a sports performance environment [64]. For an overview of the social environments’ (opponents and coaches) role in pacing and performance within the three phases of the cyclical model of SRL and how the athlete can actively use the social environment, please see Table 1.

## 5. Discussion

This study explains how self-regulatory skills influence pacing and sports performance. More importantly, it provides a theoretical rationale for the role of the social environment in the self-regulatory process of pacing and sports performance, expanding on the framework proposed by Elferink-Gemser and Hettinga [16]. This approach could partially predict the athletes’ sports behaviour and explains why racing against competitors consistently results in increased performance compared to when racing alone [8,9,10,11,12]. Thus, this study could guide athletes and coaches how to appropriately use opponents to facilitate pacing and performance acquisition. For instance, opponents could be characterised as athletes’ emotional influencers prior, during, and after the competition and alter athletes’ emotions, which are critical in pacing. More specifically, opponents act as an external motivational factor that influence athletes’ goal setting and self-efficacy [28,37]. They also decrease athletes’ cognitive loading (less depleting) and perceived exertion and facilitate a better regulation of their fatigue, afferent feedback, and pain during the trial [39,53]. Lastly, opponents could cause athletes to experience positive or negative emotions after the competitive trial and influence athletes’ affective feelings [62,63]. This is important because the ability to manage physical effort demands, tolerate unpleasant sensations, and experience positive emotions through competitions, could influence the further engagement of athletes in pacing and performance skills acquisition and the development process [62,63].

The important role of self-regulatory processes in pacing and the impact of the opponents altering athletes’ emotions has not been thoroughly explored in previous pacing-related theoretical frameworks. To be more specific, the ecological framework towards pacing thus far has been the only framework that included the role of opponents through paying attention to human–environment interactions [2,6,8]. Opponents could act as affordances for athletes and offer numerous action possibilities that enhance their motivation to attain their goals [2]. However, the ecological framework did not further expand on learning processes, or what athletes think, feel, and experience when an opponent is present. Through applying the self-regulation framework to pacing, we can now obtain a deeper understanding of their learning trajectory and the role opponents can play in this context, also allowing a role for emotions.

### Practical Applications

For all the aforementioned reasons, it is important for athletes to understand the important role of their competitors in head-to-head competitions. They have to realise how opponents can influence their goal setting (opponent as a goal-setter), their ability to monitor their actions during a competitive trial (opponent as a help-seeking factor and motivational/attentional facilitator), and their self-judgements and self-reactions processes after the trial [13,62,63]. Within this sports settings, the social environment (e.g., coaches), could observe, step in, and facilitate these intricate processes, especially when athletes are less proficient at self-regulated learning [68]. Thus, the role of the social environment could be even more beneficial in populations such us youth athletes and/or people with intellectual impairment (II) who are struggling to regulate their exercise intensity during competitive events [69,70]. Coaches should realise their crucial role in assisting athletes to develop the self-regulatory skills that a pacing and sports performance optimisation requires. It is also important for coaches to identify strategies to facilitate these processes by taking into consideration the role of opponents, as well as athletes’ abilities, traits, personalities, and needs (e.g., through social support, encouraging communication style, and positive feedback in a socially safe environment) [18,23]. For instance, at the forethought phase coaches could take into consideration opponents’ abilities and assist athletes to set challenging but realistic goals [32]. On the basis of the opponents’ profile, coaches could facilitate athletes’ sports performance skills acquisition (e.g., pacing) [5]. During the performance phase, coaches’ feedback and encouragement, with a purpose to alter athletes’ emotional state [5], could be based on opponents’ actions and behaviour (when this is appropriate). Lastly, at the self-reflection phase coaches should be aware that the opponents could positively or negatively influence athletes’ affective reactions and should adapt their coaching behaviour accordingly [64]. Due to the important coach–athlete relationship in this self-regulatory framework of pacing and performance, we suggest coach education to take into consideration the link between self-regulation, motivation, and opponents in pacing and sports [19,71]. It should also take into account the coaching suggestions that we provided above and provide coaches with a methodical approach to optimise athletes’ pacing behaviour and performance.

## 6. Conclusions

This review provided an expanded understanding of the self-regulatory basis of pacing and sports performance behaviours, building on the previously proposed model of Elferink-Gemser and Hettinga [16]. It revealed the significance of the social environment within the three phases of the cyclical model of SRL (forethought, performance, and self-reflection). More specifically, athletes can focus on their social environment (opponents) when they have to set goals and select appropriate strategies to achieve them (forethought phase), monitor and manage their actions and their emotions (performance phase), and make self-judgements and choose self-reactions (self-reflection). Coaches can facilitate these intricate processes. As the social environment is even more crucial in facilitating self-regulatory skills acquisition and improving the pacing behaviour and sports performance of athletes who are less proficient at self-regulated learning, special attention should be paid to special populations as youth athletes and athletes with II.

## Figures and Tables

**Table 1:** Overview of the social environments’ (opponents and coaches) role in pacing and performance within the three phases of the cyclical model of SRL and how the athlete can actively use the social environment.

Phases (Cyclical Model of SRL)	Self-Regulatory Skills	Opponents	Coaches	Athletes
Forethought phase	Task Analysis. Self-motivation beliefs.	Motivate athletes. Act as goal setters. Inspire athletes to plan their actions.	Motivate athletes. Set athletes’ goals. Help athletes to set their own goals and make the right decisions. Assist athletes to anticipate potential obstacles.	Set goals based on their opponents. Set goals with their coaches. Plan their activities with the coaches’ assistance.
Performance phase	Self-control. Self-observation.	Provide direct pacing and performance feedback. Act as external distractions. Facilitate athletes’ decision-making. Improve athletes’ attention. Act as help-seeking factors.	Provide motivational feedback and encouragement. Use imagery techniques. Focus on external cues (e.g., opponents).	Focus on opponents’ actions. Use opponents as pacing and performance feedback facilitators. Modify their actions based on their opponents’ behaviours.
Self-reflection phase	Self-judgements. Self-reactions.	Enable athletes to compare athletic performance (social comparisons). Influence athletes’ affective reactions.	Provide support and feedback. Regulate athletes’ affective responses based on social comparisons.	Compare themselves with their opponents. Judging competence and evaluating success and failure based on social comparisons.

## Data Availability

Not applicable.
